# Cumulative increased risk of incident type 2 diabetes mellitus with increasing triglyceride glucose index in normal-weight people: The Rural Chinese Cohort Study

**DOI:** 10.1186/s12933-017-0514-x

**Published:** 2017-03-01

**Authors:** Ming Zhang, Bingyuan Wang, Yu Liu, Xizhuo Sun, Xinping Luo, Chongjian Wang, Linlin Li, Lu Zhang, Yongcheng Ren, Yang Zhao, Junmei Zhou, Chengyi Han, Jingzhi Zhao, Dongsheng Hu

**Affiliations:** 1 Department of Preventive Medicine, Shenzhen University Health Sciences Center, 3688 Nanhai Avenue, Nanshan District, Shenzhen, 518060 Guangdong People’s Republic of China; 2 The Affiliated Luohu Hospital of Shenzhen University Health Sciences Center, Shenzhen, Guangdong People’s Republic of China; 3 Department of Epidemiology and Health Statistics, College of Public Health, Zhengzhou University, Zhengzhou, Henan People’s Republic of China; 4 Department of Prevention and Health Care, Military Hospital of Henan Province, Zhengzhou, Henan People’s Republic of China

**Keywords:** Triglyceride glucose index, Type 2 diabetes mellitus, Normal weight, Insulin resistance, Cohort study

## Abstract

**Background:**

Risk of type 2 diabetes mellitus (T2DM) is increased in metabolically obese but normal-weight people. However, we have limited knowledge of how to prevent T2DM in normal-weight people. We aimed to evaluate the association between triglyceride glucose (TyG) index and incident T2DM among normal-weight people in rural China.

**Methods:**

We included data from 5706 people with normal body mass index (BMI) (18.5–23.9 kg/m^2^) without baseline T2DM in a rural Chinese cohort followed for a median of 6.0 years. A Cox proportional-hazard model was used to assess the risk of incident T2DM by quartiles of TyG index and difference in TyG index between follow-up and baseline (TyG-D), estimating hazard ratios (HRs) and 95% confidence intervals (CIs). A generalized additive plot was used to show the nonparametric smoothed exposure–response association between risk of T2DM and TyG index as a continuous variable. TyG was calculated as ln [fasting triglyceride level (mg/dl) × fasting plasma glucose level (mg/dl)/2].

**Results:**

Risk of incident T2DM was increased with quartiles 2, 3 and 4 versus quartile 1 of TyG index (adjusted HR [aHR] 2.48 [95% CI 1.20–5.11], 3.77 [1.83–7.79], and 5.30 [2.21–12.71], *P*_trend_ < 0.001 across quartiles of TyG index). Risk of incident T2DM was increased with quartile 4 versus quartile 1 of TyG-D (aHR 3.91 [2.22–6.87]). The results were consistent when analyses were restricted to participants without baseline metabolic syndrome and impaired fasting glucose level. The generalized additive plot showed cumulative increased risk of T2DM with increasing TyG index.

**Conclusions:**

Risk of incident T2DM is increased with increasing TyG index among rural Chinese people, so the index might be an important indicator for identifying people at high risk of T2DM.

## Background

Diabetes is an important cause of mortality, morbidity, and health-system costs worldwide [1–3]. The number of diabetes deaths in the world nearly doubled between 1990 and 2010 [4]. In 2015, the International Diabetes Federation estimated that 75% of people with diabetes live in low- and middle-income countries, particularly China, which has the largest number of people with diabetes in the world (109.6 million) [5]. Additionally, rural areas of China have lower economic development than urban areas and the people have poorer knowledge of type 2 diabetes mellitus (T2DM) control and prevention [6]. In this context, T2DM has been an important public health problem in rural China.

Adiposity is the most important modifiable risk factor for T2DM [7, 8]. Although body mass index (BMI) has been widely used as a reliable and simple measure of obesity, many people with normal BMI, called “metabolically obese but normal weight (MONW)”, are characterized by a cluster of metabolic risk factors, with significantly increased incidence of T2DM in many ethnic groups [9–11]. Moreover, normal-weight people may not monitor their health indicators or take prevention measures for T2DM. Therefore, early identification of T2DM for normal-weight people is necessary. Particularly Chinese people, despite their lower absolute BMI, are more prone to visceral fat accumulation and insulin resistance (IR) than are Western populations [12, 13]. However, data are limited on the prevention of T2DM for normal-weight people in China.

Triglyceride glucose (TyG) index has been used as an inexpensive and reliable surrogate indicator to identify IR and T2DM [14–16]. The hyperinsulinemic-euglycemic clamp is considered the gold standard to define IR [16], but the technique is difficult to use in large epidemiology investigations because it is time-consuming, costly, and complex.

To our knowledge, the relationship between TyG and incident T2DM for normal-weight Chinese people has not been studied. Therefore, we designed a prospective cohort study to explore the relationship in normal-weight rural Chinese people.

## Methods

### Study population

This study was a population-based, prospective cohort study, and participants were selected by cluster randomization from eligible candidates listed in the residential registration record from the rural district in Luoyang City, Henan Province, in the middle of China, for a baseline examination during July to August of 2007 and July to August of 2008. First, Xin’an County was selected from Luoyang area, and then 2 towns were randomly selected from Xin’an County. Finally, a total of 64 villages were investigated from selected towns. Participants were excluded if they were (1) unable to answer the questionnaire; (2) unable to complete blood sampling or anthropometric or blood pressure measurements; and (3) known to have severe psychological disorders, Alzheimer’s disease, dementia, AIDS or other infectious disease. Ultimately, we recruited 20,194 participants ≥18 years old. During July to August of 2013 and July to October of 2014, all participants were invited to undergo a follow-up examination, and 17,265 participants (response rate 85.5%) were re-investigated. The other characteristics for the study population have been previously published [17, 18]. For the present study, we examined data for 8003 participants with normal weight (BMI 18.5–23.9 kg/m^2^ according to the recommendations of Working Group on Obesity of China) [19]. After excluding 430 participants with T2DM at baseline (one with gestational diabetes also), 121 participants who died due to cancer, 64 participants who died due to cardiovascular disease, 132 participants who died due to stroke, 163 participants who died due to accidents or other causes, and 1387 participants with missing lipid profile measures during follow-up, we had 5706 (3195 women) eligible for the current analyses.

### Baseline examination and data collection

Baseline data were collected at local community clinics in the participants’ residential areas. Trained research staff administered a standard questionnaire to collect information on demographic characteristics, medical history, and lifestyle risk factors. Participants who had not smoked >100 cigarettes in their lifetime were considered never-smokers; the others were considered smokers [20]. Alcohol consumption was defined as drinking alcohol >12 times during the last year. Physical activity level was classified by the International Physical Activity Questionnaire scoring protocol [21]. Family history of diabetes was defined as having at least one first-degree family member with diabetes.

Participants were asked to wear light clothes and be barefoot when measuring anthropometric indices. Body weight and height were measured to the nearest 0.1 kg and 0.1 cm, respectively, by trained investigators under standardized conditions following a standard protocol. BMI was calculated as weight in kilograms divided by the square of height in meters. With participants standing, waist circumference (WC) was measured midway between the lower edge of the costal arch and the upper edge of the iliac crest to the nearest 0.1 cm. Participants were measured twice, and the average was used. Waist-to-height ratio (WHtR) was determined by WC (cm) divided by height (cm). Central obesity was defined by WC ≥90 cm for men and ≥80 cm for women or WHtR ≥0.5 according to previous reports [22, 23]. Blood pressure was measured by using an electronic sphygmomanometer (Omron, HEM-770AFuzzy, Kyoto, Japan) after at least a 5-min rest, with participants in a seated position, according to the American Heart Association standardized protocol [24]. The measurements were repeated three times with a 30-s interval, and the average was used for analysis.

Blood samples were obtained after an overnight fast of at least 8 h. Levels of fasting plasma glucose (FPG), triglycerides (TG), total cholesterol (TC), and high-density lipoprotein cholesterol (HDL-C) were measured by using a HTACHI automatic clinical analyzer (Model 7060, Tokyo). Low-density lipoprotein cholesterol (LDL-C) level was calculated by the Friedewald formula [25]. TyG was calculated as ln[TG (mg/dl) × FPG (mg/dl)/2] [14]. The difference in TyG (TyG-D) was calculated as TyG value at the end of follow-up minus that at baseline.

T2DM was defined as FPG ≥7.0 mmol/l and/or current treatment with anti-diabetes medication according to the China guideline for type 2 diabetes [26].

### Follow-up examination

Study participants or their proxies at their current address underwent in-depth interviews to ascertain disease status and vital information; hospital records and death certificates were obtained as well. Data collection at follow-up was the same as at baseline.

### Statistical analysis

All continuous variables are described with median (interquartile range) because of skewed distribution. Categorical variables are presented as number (percentage). Study participants were classified by four TyG quartiles. The linear trend for baseline characteristics was tested by linear regression for continuous variables and logistic regression for categorical variables.

A Cox proportional-hazard regression model was used to estimate hazard ratios (HRs) and 95% confidence intervals (CIs) for incident T2DM by quartiles of TyG and TyG-D, with the lowest quartile as the reference. Besides the unadjusted model, 3 other models were fitted: model 1, controlling for gender, age, family history of diabetes, and WC; model 2, additionally adjusted for education level, marital status, smoking, alcohol consumption, and physical activity; model 3, adjusted for variables included in model 2 plus systolic blood pressure (SBP), diastolic blood pressure (DBP) and TC, HDL-C, and LDL-C levels (all of these factors related to FPG level). The linear trends across TyG and TyG-D quartiles were evaluated by a median value within each quartile as a continuous variable. The receiver operating characteristic (ROC) curve and area under the ROC curve (AUC) were used to compare the ability of baseline TyG index, WC and WHtR to predict risk of T2DM at follow-up.

The cumulative incidence of T2DM and cumulative incidence curves by TyG quartile were examined by competing risk regression analysis. Generalized additive models were used to test for nonlinearity in the analysis of risk of T2DM by TyG index with adjustment for potential confounding variables (gender, age, and family history of diabetes, WC, SBP, DBP, and TC, HDL-C and LDL-C levels).

Sensitivity analysis was conducted to assess the robustness of the results by rerunning all the models excluding 899 participants with metabolic syndrome (Third Report of the National Cholesterol Education Program’s Adult Treatment Panel criteria components modified for WC cutoff according to World Health Organization Asia Pacific guidelines) [27] and 202 participants with impaired FPG level (FPG 6.1–7.0 mmol/l) [26, 28] at baseline. ROC analysis, competing risk regression analysis and generalized additive model construction involved use of STATA v12.0 (STATA Corp, College Station, TX, USA) and other analyses involved SAS 9.1 (SAS Inst., Cary, NC, USA). Two-sided *P* < 0.05 was considered statistically significant.

## Results

### Baseline characteristics of study participants

Data were analyzed for 5706 normal-weight people (median age 51 years [interquartile range 40–60]). After 34,246.00 person-years of follow-up, T2DM developed in 194 participants; the overall incidence of T2DM was 5.66/1000 person-years. Age, BMI, WC, WHtR, SBP, and DBP were increased by quartiles of TyG index (all *P*_trend_ < 0.001) (Table 1). Compared with participants with quartile 1 of TyG index, those with higher quartiles were more frequently older, women, with less education and less physically active. Marital status, smoking, alcohol consumption, and family history of diabetes were similar across TyG quartiles. FPG, TC, TG, and LDL-C levels were increased and HDL-C level was decreased with increasing TyG quartile (all *P*_trend_ < 0.001).

**Table 1:** Baseline characteristics of study participants stratified by quartiles of triglyceride glucose (TyG) index

Variables	TyG index quartiles				*P*_trend_
	Quartile 1 (<8.16) n = 1423	Quartile 2 (8.16–8.47) n = 1422	Quartile 3 (8.48–8.81) n = 1429	Quartile 4 (≥8.82) n = 1424	
Age (years)	44.00 (36.00–56.00)	51.00 (40.00–60.00)	52.00 (43.00–61.00)	54.00 (44.00–62.00)	*<0.001*
Men	618 (43.43)	679 (47.75)	638 (44.65)	571 (40.10)	*0.03*
High school or above	156 (10.96)	180 (12.66)	140 (9.80)	121 (8.50)	*<0.01*
Married/cohabiting	1274 (89.53)	1294 (91.06)	1306 (91.39)	1303 (91.50)	0.07
Smoking	436 (30.64)	495 (34.81)	456 (31.91)	411 (28.86)	0.14
Drinking	160 (11.24)	166 (11.67)	179 (12.53)	161 (11.31)	0.78
Physical activity					
Low	793 (55.73)	777 (54.64)	755 (52.83)	692 (48.60)	*<0.001*
Middle	282 (19.82)	279 (19.62)	290 (20.29)	321 (22.54)	
High	348 (24.46)	366 (25.74)	384 (26.87)	411 (28.86)	
Family history of diabetes^a^	60 (5.24)	62 (5.36)	56 (4.83)	70 (6.03)	0.54
BMI (kg/m^2^)	21.53 (20.39–22.60)	21.57 (20.32–22.73)	21.94 (20.69–23.01)	22.35 (21.20–23.24)	*<0.001*
WC (cm)	73.75 (70.25–78.00)	74.50 (70.75–78.75)	76.03 (71.93–80.50)	78.25 (74.10–82.15)	*<0.001*
WHtR	0.46 (0.44–0.49)	0.47 (0.44–0.49)	0.48 (0.45–0.51)	0.49 (0.47–0.52)	*<0.001*
FPG (mmol/l)	5.00 (4.71–5.31)	5.18 (4.86–5.48)	5.27 (4.98–5.62)	5.43 (5.14–5.81)	*<0.001*
SBP (mmHg)	113.67 (105.33–126.00)	118.00 (108.00–130.67)	119.00 (109.33–131.67)	123.00 (112.33–136.67)	*<0.001*
DBP (mmHg)	71.67 (66.33–78.33)	74.00 (68.00–81.00)	74.67 (69.00–81.67)	77.00 (70.00–84.00)	*<0.001*
TC (mmol/l)	3.79 (3.38–4.27)	4.10 (3.65–4.64)	4.37 (3.85–4.96)	4.72 (4.09–5.28)	*<0.001*
TG (mmol/l)	0.69 (0.59–0.78)	1.01 (0.92–1.10)	1.35 (1.23–1.48)	2.03 (1.78–2.56)	*<0.001*
HDL-C (mmol/l)	1.25 (1.10–1.44)	1.21 (1.04–1.39)	1.17 (1.02–1.36)	1.10 (0.95–1.25)	*<0.001*
LDL-C (mmol/l)	2.20 (1.90–2.60)	2.40 (2.00–2.90)	2.60 (2.10–3.10)	2.60 (2.10–3.10)	*<0.001*
TyG index	7.93 (7.77–8.06)	8.33 (8.25–8.41)	8.64 (8.57–8.73)	9.08 (8.94–9.31)	*<0.001*

Significant *P* values (*P* < 0.05) are in italics

Data are median (interquartile range) or no. (%)

*BMI* body mass index, *WC* waist circumference, *WHtR* waist-to-height ratio, *FPG* fasting plasma glucose, *SBP* systolic blood pressure, *DBP* diastolic blood pressure, *TC* total cholesterol, *TG* triglyceride, *HDL-C* high-density lipoprotein cholesterol, *LDL-C* low-density lipoprotein cholesterol, *TyG* triglyceride glucose

^a^Partial data deletion

### Risk of incident T2DM by quartiles of TyG and TyG-D

**Fig. 1:**
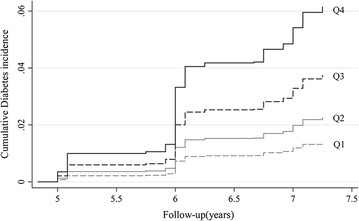
Cumulative incidence of type 2 diabetes mellitus (T2DM) by quartiles of triglyceride glucose (TyG) index. *Gray dash line* quartile 1; *gray solid line* quartile 2; *black dash line* quartile 3; *black solid line* quartile 4

With increasing TyG quartile, T2DM incidence increased substantially for normal-weight people, reaching an incidence of 10.48/1000 person-years for quartile 4 of TyG index (Table 2). On competing risk analysis, the cumulative risk of T2DM increased over time by baseline TyG quartile (Fig. 1) and remained significant even after adjustment for potential confounding factors (adjusted HR [aHR] [model 3] 2.48 [95% CI 1.20–5.11], 3.77 [1.83–7.79], and 5.30 [2.21–12.71] for quartiles 2, 3 and 4 versus quartile 1 of TyG index) (Table 2). Moreover, risk of incident T2DM was increased with increasing TyG quartile (*P*_trend_ < 0.001). Risk of incident T2DM was increased with quartile 4 versus quartile 1 of TyG-D index (aHR 3.91 [2.22–6.87]; model 3; Table 2) (*P*_trend_ < 0.001).

**Table 2:** Risk of incident type 2 diabetes mellitus (T2DM) by quartiles of TyG and TyG-D

	Quartile 1	Quartile 2	Quartile 3	Quartile 4	*P*_trend_
TyG					
Range	<8.16	8.16 to 8.47	8.48 to 8.81	≥8.82	
No. of cases	18	35	52	88	
No. of person-years	8740.50	8526.83	8529.25	8399.50	
Incidence rate^a^	2.06	4.10	6.10	10.48	<0.001
Unadjusted model	1 (ref)	2.36 (1.33–4.16)	3.58 (2.09–6.12)	6.30 (3.78–10.50)	<0.001
Multivariable adjusted model 1^b^	1 (ref)	2.38 (1.17–4.85)	3.71 (1.89–7.29)	5.54 (2.87–10.71)	<0.001
Multivariable adjusted model 2^c^	1 (ref)	2.49 (1.22–5.08)	3.89 (1.98–7.65)	5.69 (2.94–11.01)	<0.001
Multivariable adjusted model 3^d^	1 (ref)	2.48 (1.20–5.11)	3.77 (1.83–7.79)	5.30 (2.21–12.71)	<0.001
TyG-D					
Range	<−0.27	−0.27 to 0	0.01 to 0.31	≥0.32	
No. of cases	41	39	30	83	
No. of person-years	8520.00	9747.08	7373.00	8556.00	
Incidence rate^a^	4.81	4.00	4.07	9.70	<0.001
Unadjusted model	1 (ref)	0.95 (0.58–1.55)	1.15 (0.68–1.93)	2.19 (1.41–3.39)	<0.001
Multivariable adjusted model 1^b^	1 (ref)	0.92 (0.52–1.63)	1.09 (0.59–2.03)	2.51 (1.50–4.19)	<0.001
Multivariable adjusted model 2^c^	1 (ref)	0.92 (0.52–1.64)	1.10 (0.59–2.05)	2.52 (1.51–4.22)	<0.001
Multivariable adjusted model 3^d^	1 (ref)	1.14 (0.63–2.09)	1.54 (0.80–2.99)	3.91 (2.22–6.87)	<0.001

Data are hazard ratios (HRs) and 95% confidence intervals (95% CIs)

*TyG* triglyceride glucose index, *TyG-D* difference in TyG value at the end of follow-up minus that at baseline

^a^Per 1000 person-years

^b^Adjusted for gender, age, family history of diabetes and WC

^c^Adjusted for variables in ^b^ as well as education level, marital status, smoking, alcohol consumption and physical activity

^d^Adjusted for variables in ^c^ as well as SBP, DBP and TC, HDL-C and LDL-C levels

**Fig. 2:**
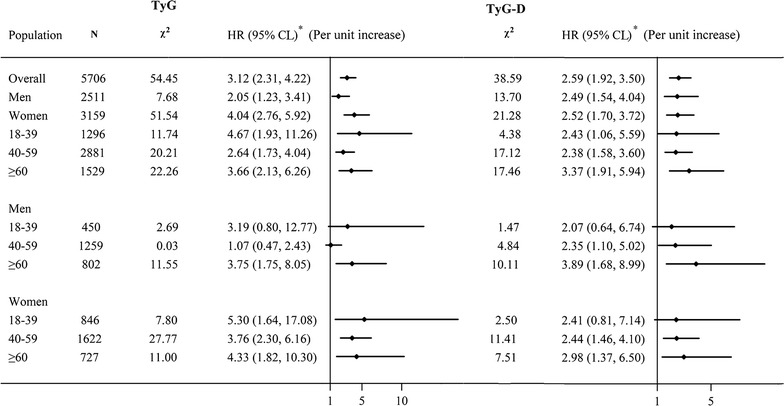
Association of T2DM and TyG index or TyG-D by sex and/or age groups. Data are hazard ratios (HRs) and 95% confidence limits (95% CLs). *T2DM* type 2 diabetes mellitus, *TyG index* triglyceride glucose index, *TyG-D* difference in TyG value at the end of follow-up minus that at baseline. *Asterisk* adjusted for gender, age, education level, marital status, smoking, alcohol consumption, physical activity, family history of diabetes and waist circumference (WC)

Risk of incident T2DM was increased with per-unit increase in TyG index for both genders and all age groups (Fig. 2). With per-unit increase of baseline TyG value, the risk of T2DM increased twofold for men and fourfold for women (aHR 2.05 [95% CI 1.23–3.41] and 4.04 [2.76–5.92], respectively; model 2). Risk of incident T2DM was increased with per-unit increase in TyG index for older men (aHR, 3.75 [1.75–8.05]) and women of all age groups. The risk with high TyG-D value was significantly associated with middle and older age groups for both men and women. These results were consistent with abdominal fat distribution for different sex/age groups (Table 3). With WC criteria, the prevalence of central obesity was low for men of all age groups (1.62–1.91%), but with WHtR criteria, it was highest for older men (23.82%) as compared with young and middle-aged men. Regardless of the criteria used, women had the high prevalence of central obesity for all age groups.

**Table 3:** The prevalence of central obesity at baseline based on WC and WHtR criteria by age and gender

	N	Defined by WC		Defined by WHtR	
		No. of individuals	Prevalence (%)	No. of individuals	Prevalence (%)
Men					
18–39	450	8	1.78	40	8.89
40–59	1259	24	1.91	203	16.14
≥60	802	13	1.62	191	23.82
Women					
18–39	846	100	11.82	129	15.25
40–59	1622	307	18.49	558	34.42
≥60	727	241	33.20	424	58.40

*WC* waist circumference, *WHtR* waist-to-height ratio

### ROC analyses for TyG, WC, and WHtR to predict the incident risk of T2DM

**Fig. 3:**
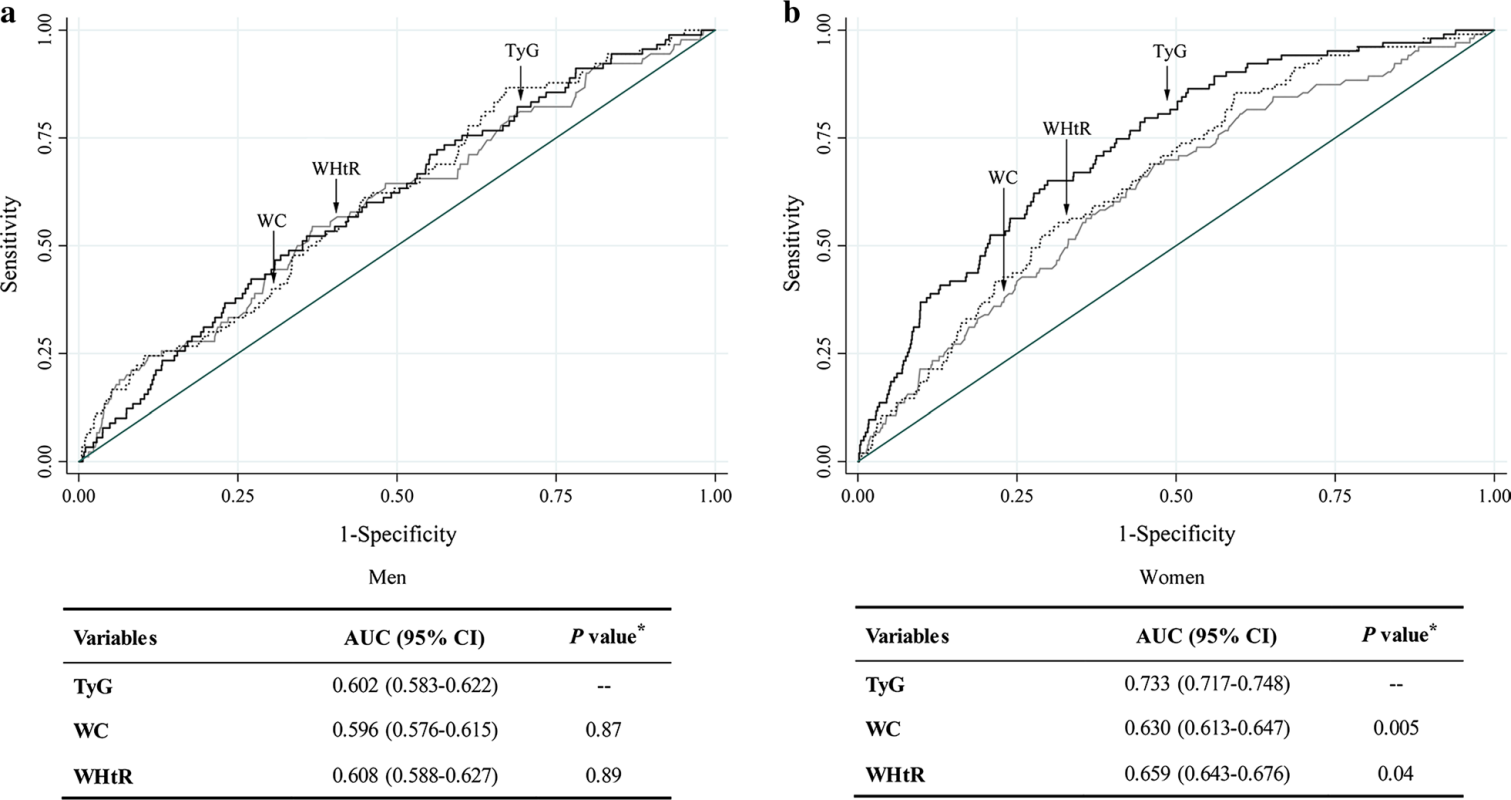
ROC curves for TyG, WC, and WHtR for predicting T2DM among men (**a**), and women (**b**). *ROC* receiver operating characteristic, *TyG index* triglyceride glucose index, *WC* waist circumference, *WHtR* waist-to-height ratio, *T2DM* type 2 diabetes mellitus. *Solid black line* TyG; *solid gray line*,WC; *dotted black line* WHtR. *Asterisk* compared with TyG

For men, the AUC for TyG, WC, and WHtR was 0.602 (0.583–0.622), 0.596 (0.576–0.615), and 0.608 (0.588–0.627), respectively (Fig. 3). The AUC for TyG index, WC, and WHtR was larger for women than men: 0.733 (0.717–0.748), 0.630 (0.613–0.647), and 0.659 (0.643–0.676), respectively, with significant differences between AUCs for TyG index and WC (*P* = 0.005) and TyG index and WHtR (*P* = 0.04). The best TyG value for diagnosis of T2DM was 8.64 (sensitivity 0.522, specificity 0.642) for men and 8.76 (sensitivity 0.650, specificity 0.702) for women.

### Generalized additive plot for TyG and T2DM risk

**Fig. 4:**
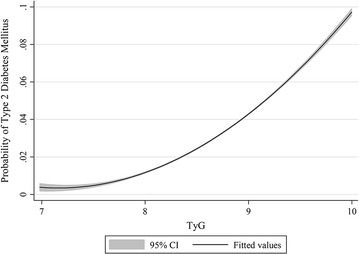
Generalized additive model plot for risk of T2DM and TyG index. Adjusted for gender, age, family history of diabetes, waist circumference (WC), systolic blood pressure (SBP), diastolic blood pressure (DBP), total cholesterol (TC), high-density lipoprotein cholesterol (HDL-C) and low-density lipoprotein cholesterol (LDL-C). *T2DM* type 2 diabetes mellitus, *TyG index* triglyceride glucose index

Figure 4 shows the nonparametric smoothed exposure–response relationship between TyG index and T2DM risk (TyG index as a continuous variable). The function for TyG index showed significant nonlinearity (*P* < 0.001) with T2DM risk, and the slope increased with increasing TyG index.

### Results of sensitivity analysis

**Fig. 5:**
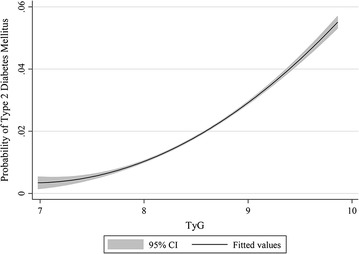
Generalized additive model plot for T2DM and TyG with participants without baseline IFG and MetS. *IFG* impaired fasting glucose, *MetS* metabolic syndrome, *T2DM* type 2 diabetes mellitus, *TyG index* triglyceride glucose index. Adjusted for gender, age, family history of diabetes, waist circumference (WC), systolic blood pressure (SBP), diastolic blood pressure (DBP), total cholesterol (TC), high-density lipoprotein cholesterol (HDL-C) and low-density lipoprotein cholesterol (LDL-C)

The main results of the current study were consistent when analyses were restricted to participants without metabolic syndrome and impaired FPG level at baseline (Table 4). After 27,801.75 person-years of follow-up, the T2DM incidence was 3.45/1000 person-years (96 cases) (data not shown). Risk of T2DM was increased by 4.29-fold (95% CI 1.72–10.67) and 5.88-fold (95% CI 2.06–16.76) with quartiles 3 and 4 versus quartile 1 of TyG, respectively (model 3). A generalized additive model plot showed that TyG index had significant nonlinearity with T2DM risk (*P* = 0.25) (Fig. 5), but the association was almost linear after excluding participants with metabolic syndrome and impaired FPG level at baseline.

**Table 4:** Risk of incident T2DM for participants without metabolic syndrome and impaired FPG level at baseline

	Quartile 1	Quartile 2	Quartile 3	Quartile 4	*P*_trend_
TyG					
Range	<8.09	8.09 to 8.39	8.40 to 8.68	≥8.69	
No. of cases	12	15	32	37	
No. of person-years	7078.67	6945.58	6914.00	6832.42	
Incidence rate^a^	1.70	2.16	4.63	5.42	<0.001
Unadjusted model	1 (ref)	1.42 (0.67–3.04)	3.10 (1.60–6.03)	3.75 (1.95–7.23)	<0.001
Multivariable adjusted model 1^b^	1 (ref)	1.19 (0.43–3.30)	3.50 (1.50–8.16)	4.36 (1.89–10.05)	<0.001
Multivariable adjusted model 2^c^	1 (ref)	1.25 (0.45–3.46)	3.68 (1.57–8.60)	4.54 (1.97–10.47)	<0.001
Multivariable adjusted model 3^d^	1 (ref)	1.34 (0.48–3.76)	4.29 (1.72–10.67)	5.88 (2.06–16.76)	<0.001
TyG-D					
Range	<−0.23	−0.23 to 0	0.01 to 0.35	≥0.35	
No. of cases	17	14	20	45	
No. of person-years	6912.75	7101.50	6797.33	6959.08	
Incidence rate^a^	2.46	1.97	2.94	6.47	<0.001
Unadjusted model	1 (ref)	0.78 (0.37–1.64)	1.37 (0.69–2.70)	2.40 (1.31–4.39)	<0.001
Multivariable adjusted model 1^b^	1 (ref)	0.78 (0.33–1.84)	0.97 (0.41–2.28)	2.86 (1.40–5.84)	<0.001
Multivariable adjusted model 2^c^	1 (ref)	0.80 (0.34–1.90)	0.98 (0.42–2.32)	2.90 (1.42–5.93)	<0.001
Multivariable adjusted model 3^d^	1 (ref)	0.99 (0.42–2.36)	1.43 (0.60–3.43)	4.56 (2.14–9.70)	<0.001

Data are hazard ratios (HRs) and 95% confidence intervals (95% CIs)

^a^Per 1000 person-years

^b^Adjusted for gender, age, family history of diabetes and WC

^c^Adjusted for variables in ^b^ as well as education level, marital status, smoking, alcohol consumption and physical activity

^d^Adjusted for variables in ^c^ as well as SBP, DBP and TC, HDL-C and LDL-C levels

## Discussion

This population-based prospective cohort study suggests a cumulative increased risk of incident T2DM with increasing TyG index in rural Chinese people with normal weight. These findings appeared to extend the risk of T2DM to normal-weight participants without baseline metabolic syndrome and impaired FPG level. To our knowledge, this study is the first to demonstrate a significant association between TyG index, a biomarker of IR, and incident T2DM in rural normal-weight Chinese people. This association was particularly evident for women of all age groups and for older men, which may due to the abdominal fat distribution for different sex/age groups in our population (Table 3). In addition, women tend to have higher hepatocellular lipids, both fasting and after glucose and lipid loading as compared with men [29, 30]. As people age, visceral adiposity tissue is significantly increased for both genders, which can increase the risk of T2DM [29]. As well, risk of incident T2DM was increased with high TyG value at follow-up for normal-weight people, which agrees with results from Korea [31].

Many observational studies demonstrated that more than 30% of normal-weight people have metabolic abnormalities (MONW-like phenotype), including abdominal fat accumulation and IR [11, 32, 33]. Prospective cohort studies have demonstrated increased risk of T2DM in people with MONW in different ethnic groups [11, 33, 34]. Data from the Whitehall II study showed that the incidence of T2DM for MONW people was 12.46/1000 person-years, and the risk of incident T2DM was more than threefold increased as compared with metabolically healthy and normal weight people [11]. Similar results were observed in an Asian population [33]. However, measures to prevent T2DM in normal-weight people are lacking. Our results provide evidence that the TyG index can be an important indicator for predicting T2DM among normal-weight people.

Decreased β-cell function and IR are considered central events in the development of T2DM [35]. Islets have intrinsically low antioxidant enzyme defenses; elevated glucose concentrations increasing levels of reactive oxygen species in β cells have toxic effects on β cells, further leading to IR and T2DM [36]. Under conditions such as lipodystrophy, the lipolysis is enhanced, and prolonged fatty-acid exposure in β cells can decrease glucose-induced insulin secretion, impair insulin gene expression, and increase cell death [37–39]. As well, TG overload in islets interferes with glucose metabolism and impairs the function of β cells [40]. When both fatty acids and glucose are elevated, the accumulation of metabolites derived from fatty-acid esterification impairs β-cell function [36]. Changes in TG and HDL-C have a unidirectional relationship with peripheral IR, which provides evidence for the early prevention of IR by improving dyslipidemia [41]. Studies of diabetes patients added that the capacity for insulin secretion and IR are closely affected by TG and FPG levels [42–44], which was also confirmed in an intervention study [45]. After appropriate treatment of high TG and high FPG level by n-3 fatty acids in patients with impaired glucose metabolism, the ability of insulin secretion was improved [45].

Visceral adipose tissue is associated with increased cytokine production and IR [46]. Epidemiology studies have found BMI inadequate to define obesity; it cannot adequately discriminate between fat mass and lean tissues nor identify regional body fat distribution [47–49]. A population-based cross-sectional study of Chinese people showed T2DM significantly associated with central obesity among adults with normal BMI [47]. Another study of a Japanese population demonstrated risk of prediabetes with upper-normal WC for people with normal WC and BMI after adjusting for BMI [48]. This situation may explain why some people with normal BMI show significantly increased risk of T2DM. Another explanation may be inherited genetic factors. The genetic variation near insulin receptor substrate 1 (*IRS1*) is associated with low body fat but also impaired metabolic profile, including decreased subcutaneous-to-visceral fat ratio, increased insulin resistance, dyslipidemia, risk of diabetes and coronary artery disease, and decreased adiponectin level [49].

TyG index, a biomarker related to IR [50, 51], can be used in clinical practice because measuring TG and glucose is inexpensive and routine. As compared with the hyperinsulinemic-euglycemic clamp, the TyG index showed high sensitivity (96.5%) and specificity (85.0%) for diagnosis of insulin resistance in Mexican people [52]. The validation study was conducted in a Brazilian population, and TyG index performed better than homeostatic model assessment for measuring IR in clinical practice [16]. In a 4-year retrospective longitudinal study of Korean people, high baseline TyG index was related to T2DM development regardless of obesity status [53]. Therefore, the TyG index may be clinically important for preventing T2DM. However, compared with other routine biomarkers, the ability of TyG to identify IR or T2DM was controversial in different ethnic groups. TyG index did not improve diabetes prediction as compared with FPG, 1-h plasma glucose, and 2-h plasma glucose in an Iranian population [54]. However, in a large European population, TyG index was a better predictor than FPG or TG level of T2DM development in normoglycemic people [15]. Compared with obesity-related markers (BMI and WC) and single lipid markers (TC, TG, HDL-C, and LDL-C), TyG index had a larger AUC for identifying IR and diabetes but lower than the markers combining TyG and obesity (TyG-WC and TyG-BMI) for Chinese people [55, 56]. In the present study, the ability of TyG index to predict incident T2DM was similar to WC and WHtR for men but better than WC or WHtR for women.

For normal-weight people, only one study conducted in rural Korea reported that TyG index was useful for predicting the incidence of T2DM in both men and women [33]. Hence, the index needs to be validated to identify T2DM in normal-weight people in different ethnic groups. In our rural Chinese normal-weight population, risk of incident T2DM increased with increasing TyG value, and the association was almost linear for people with baseline normal FPG level and metabolic health.

The strengths of the study include its prospective design, large community-based sample of men and women across a broad age spectrum, high rates of participation, standardized high-quality clinical and laboratory procedures, and adjustment for a large number of potential confounders. We also conducted a sensitivity analysis to assess the robustness of the association between TyG index and incident T2DM risk by rerunning all the models excluding participants with metabolic syndrome and impaired FPG level at baseline.

However, the study has several limitations. First, we did not use 2-h oral glucose tolerance test and glycated haemoglobin (HbA_1c_) for diagnosing T2DM, so we may have underestimated the incidence of T2DM. As well, most of our participants were not clear about whether they used lipid-lowering medications, so we did not include this variable in the analysis. Finally, because all participants were rural Chinese people, the applicability and utility of TyG index for predicting T2DM in normal-weight people should be confirmed in other ethnic populations.

## Conclusions

Our study suggests an increased risk of T2DM with increasing TyG index for normal-weight people in a Chinese rural cohort, particularly women. TyG may be useful for predicting T2DM among normal-weight people.

## Abbreviations

AUC: area under the receiver operation characteristic curve
BMI: body mass index
CI: confidence interval
DBP: diastolic blood pressure
FPG: fasting plasma glucose
HDL-C: high-density lipoprotein cholesterol
HR: hazard ratio
IR: insulin resistance
MONW: metabolically obese but normal weight
LDL-C: low-density lipoprotein cholesterol
ROC: receiver operating characteristic
SBP: systolic blood pressure
T2DM: type 2 diabetes mellitus
TC: total cholesterol
TG: triglyceride
TyG: triglyceride glucose
TyG-D: difference in TyG value at the end of follow-up minus that at baseline
WC: waist circumference
WHtR: waist-to-height ratio

## References

[1] Global Burden of Metabolic Risk Factors for Chronic Diseases C (2014). Cardiovascular disease, chronic kidney disease, and diabetes mortality burden of cardiometabolic risk factors from 1980 to 2010: a comparative risk assessment. Lancet Diabetes Endocrinol.

[2] Seuring T, Archangelidi O, Suhrcke M (2015). The economic costs of type 2 diabetes: a global systematic review. Pharmacoeconomics.

[3] Shaw JE, Sicree RA, Zimmet PZ (2010). Global estimates of the prevalence of diabetes for 2010 and 2030. Diabetes Res Clin Pract.

[4] Lozano R, Naghavi M, Foreman K (2012). Global and regional mortality from 235 causes of death for 20 age groups in 1990 and 2010: a systematic analysis for the Global Burden of Disease Study 2010. Lancet.

[5] Federation ID. IDF diabetes atlas. 7th ed. 2015. http://www.diabetesatlas.org/key-messages.html. Accessed 17 Feb 2017.

[6] Xu Y, Wang L, He J (2013). Prevalence and control of diabetes in Chinese adults. JAMA.

[7] Singh GM, Danaei G, Farzadfar F (2013). The age-specific quantitative effects of metabolic risk factors on cardiovascular diseases and diabetes: a pooled analysis. PLoS ONE.

[8] Prospective Studies C, Whitlock G, Lewington S (2009). Body-mass index and cause-specific mortality in 900 000 adults: collaborative analyses of 57 prospective studies. Lancet.

[9] Karelis AD, St-Pierre DH, Conus F, Rabasa-Lhoret R, Poehlman ET (2004). Metabolic and body composition factors in subgroups of obesity: what do we know?. J Clin Endocrinol Metab.

[10] Soriguer F, Gutierrez-Repiso C, Rubio-Martin E (2013). Metabolically healthy but obese, a matter of time? Findings from the prospective Pizarra study. J Clin Endocrinol Metab.

[11] Hinnouho GM, Czernichow S, Dugravot A (2015). Metabolically healthy obesity and the risk of cardiovascular disease and type 2 diabetes: the Whitehall II cohort study. Eur Heart J.

[12] Nazare JA, Smith JD, Borel AL (2012). Ethnic influences on the relations between abdominal subcutaneous and visceral adiposity, liver fat, and cardiometabolic risk profile: the International Study of Prediction of Intra-Abdominal Adiposity and Its Relationship With Cardiometabolic Risk/Intra-Abdominal Adiposity. Am J Clin Nutr.

[13] Gao H, Salim A, Lee J, Tai ES, van Dam RM (2012). Can body fat distribution, adiponectin levels and inflammation explain differences in insulin resistance between ethnic Chinese, Malays and Asian Indians?. Int J Obes.

[14] Simental-Mendia LE, Rodriguez-Moran M, Guerrero-Romero F (2008). The product of fasting glucose and triglycerides as surrogate for identifying insulin resistance in apparently healthy subjects. Metab Syndr Relat Disord.

[15] Navarro-Gonzalez D, Sanchez-Inigo L, Pastrana-Delgado J, Fernandez-Montero A, Martinez JA (2016). Triglyceride-glucose index (TyG index) in comparison with fasting plasma glucose improved diabetes prediction in patients with normal fasting glucose: the vascular-metabolic CUN cohort. Prev Med.

[16] Vasques AC, Novaes FS, de Oliveira Mda S (2011). TyG index performs better than HOMA in a Brazilian population: a hyperglycemic clamp validated study. Diabetes Res Clin Pract.

[17] Zhao Y, Zhang M, Luo X (2016). Association of obesity categories and high blood pressure in a rural adult Chinese population. J Hum Hypertens.

[18] Li YQ, Sun CQ, Li LL (2014). Resting heart rate as a marker for identifying the risk of undiagnosed type 2 diabetes mellitus: a cross-sectional survey. BMC Public Health.

[19] Zhou B, Coorperative Meta-Analysis Group Of China Obesity Task F (2002). Predictive values of body mass index and waist circumference to risk factors of related diseases in Chinese adult population. Zhonghua Liu Xing Bing Xue Za Zhi.

[20] Wildman RP, Muntner P, Reynolds K (2008). The obese without cardiometabolic risk factor clustering and the normal weight with cardiometabolic risk factor clustering: prevalence and correlates of 2 phenotypes among the US population (NHANES 1999–2004). Arch Intern Med.

[21] Craig CL, Marshall AL, Sjostrom M (2003). International physical activity questionnaire: 12-country reliability and validity. Med Sci Sports Exerc.

[22] Srinivasan SR, Wang R, Chen W, Wei CY, Xu J, Berenson GS (2009). Utility of waist-to-height ratio in detecting central obesity and related adverse cardiovascular risk profile among normal weight younger adults (from the Bogalusa Heart Study). Am J Cardiol.

[23] He YH, Jiang GX, Yang Y (2009). Obesity and its associations with hypertension and type 2 diabetes among Chinese adults age 40 years and over. Nutrition.

[24] Perloff D, Grim C, Flack J (1993). Human blood pressure determination by sphygmomanometry. Circulation.

[25] Bairaktari E, Hatzidimou K, Tzallas C (2000). Estimation of LDL cholesterol based on the Friedewald formula and on apo B levels. Clin Biochem.

[26] Weng J, Ji L, Jia W (2016). Standards of care for type 2 diabetes in China. Diabetes Metab Res Rev.

[27] National Cholesterol Education Program Expert Panel on Detection E, Treatment of High Blood Cholesterol in A (2002). Third report of the national cholesterol education program (NCEP) expert panel on detection, evaluation, and treatment of high blood cholesterol in adults (adult treatment panel III) final report. Circulation.

[28] Society CD (2014). China guideline for type 2 diabetes. Chin J Endocrinol Metab..

[29] Machann J, Thamer C, Schnoedt B (2005). Age and gender related effects on adipose tissue compartments of subjects with increased risk for type 2 diabetes: a whole body MRI/MRS study. MAGMA.

[30] Greenman Y, Golani N, Gilad S, Yaron M, Limor R, Stern N (2004). Ghrelin secretion is modulated in a nutrient- and gender-specific manner. Clin Endocrinol.

[31] Lee SH, Yang HK, Ha HS (2015). Changes in metabolic health status over time and risk of developing type 2 diabetes: a prospective cohort study. Medicine.

[32] Wang B, Zhuang R, Luo X (2015). Prevalence of metabolically healthy obese and metabolically obese but normal weight in adults worldwide: a meta-analysis. Horm Metab Res.

[33] Lee SH, Han K, Yang HK (2015). A novel criterion for identifying metabolically obese but normal weight individuals using the product of triglycerides and glucose. Nutr Diabetes.

[34] Kim NH, Seo JA, Cho H (2016). Risk of the development of diabetes and cardiovascular disease in metabolically healthy obese people: The Korean Genome and Epidemiology Study. Medicine.

[35] Alejandro EU, Gregg B, Blandino-Rosano M, Cras-Meneur C, Bernal-Mizrachi E (2015). Natural history of beta-cell adaptation and failure in type 2 diabetes. Mol Aspects Med.

[36] Robertson RP, Harmon J, Tran PO, Poitout V (2004). Beta-cell glucose toxicity, lipotoxicity, and chronic oxidative stress in type 2 diabetes. Diabetes.

[37] Mason TM, Goh T, Tchipashvili V (1999). Prolonged elevation of plasma free fatty acids desensitizes the insulin secretory response to glucose in vivo in rats. Diabetes.

[38] Jacqueminet S, Briaud I, Rouault C, Reach G, Poitout V (2000). Inhibition of insulin gene expression by long-term exposure of pancreatic beta cells to palmitate is dependent on the presence of a stimulatory glucose concentration. Metabolism.

[39] Maedler K, Spinas GA, Dyntar D, Moritz W, Kaiser N, Donath MY (2001). Distinct effects of saturated and monounsaturated fatty acids on beta-cell turnover and function. Diabetes.

[40] Unger RH (1995). Lipotoxicity in the pathogenesis of obesity-dependent NIDDM. Genetic and clinical implications. Diabetes.

[41] Han T, Cheng Y, Tian S (2016). Changes in triglycerides and high-density lipoprotein cholesterol may precede peripheral insulin resistance, with 2-h insulin partially mediating this unidirectional relationship: a prospective cohort study. Cardiovasc Diabetol.

[42] Riboldi BP, Luft VC, de Castilhos CD (2015). Glucose and triglyceride excursions following a standardized meal in individuals with diabetes: ELSA-Brasil study. Cardiovasc Diabetol.

[43] Rosenblit PD (2016). Common medications used by patients with type 2 diabetes mellitus: what are their effects on the lipid profile?. Cardiovasc Diabetol.

[44] Leon-Acuna A, Alcala-Diaz JF, Delgado-Lista J (2016). Hepatic insulin resistance both in prediabetic and diabetic patients determines postprandial lipoprotein metabolism: from the CORDIOPREV study. Cardiovasc Diabetol.

[45] Sawada T, Tsubata H, Hashimoto N (2016). Effects of 6-month eicosapentaenoic acid treatment on postprandial hyperglycemia, hyperlipidemia, insulin secretion ability, and concomitant endothelial dysfunction among newly-diagnosed impaired glucose metabolism patients with coronary artery disease. An open label, single blinded, prospective randomized controlled trial. Cardiovasc Diabetol.

[46] McLaughlin T, Lamendola C, Liu A, Abbasi F (2011). Preferential fat deposition in subcutaneous versus visceral depots is associated with insulin sensitivity. J Clin Endocrinol Metab.

[47] Zhang P, Wang R, Gao C (2016). Prevalence of central obesity among adults with normal BMI and its association with metabolic diseases in Northeast China. PLoS ONE.

[48] Okada R, Yasuda Y, Tsushita K, Wakai K, Hamajima N, Matsuo S (2016). Upper-normal waist circumference is a risk marker for metabolic syndrome in normal-weight subjects. Nutr Metab Cardiovasc Dis.

[49] Kilpelainen TO, Zillikens MC, Stancakova A (2011). Genetic variation near IRS1 associates with reduced adiposity and an impaired metabolic profile. Nat Genet.

[50] Guerrero-Romero F, Villalobos-Molina R, Jimenez-Flores JR (2016). Fasting triglycerides and glucose index as a diagnostic test for insulin resistance in young adults. Arch Med Res.

[51] Mohd Nor NS, Lee S, Bacha F, Tfayli H, Arslanian S (2016). Triglyceride glucose index as a surrogate measure of insulin sensitivity in obese adolescents with normoglycemia, prediabetes, and type 2 diabetes mellitus: comparison with the hyperinsulinemic-euglycemic clamp. Pediatr Diabetes.

[52] Guerrero-Romero F, Simental-Mendia LE, Gonzalez-Ortiz M (2010). The product of triglycerides and glucose, a simple measure of insulin sensitivity. Comparison with the euglycemic-hyperinsulinemic clamp. J Clin Endocrinol Metab.

[53] Lee DY, Lee ES, Kim JH (2016). Predictive value of triglyceride glucose index for the risk of incident diabetes: a 4-year Retrospective Longitudinal Study. PLoS ONE.

[54] Janghorbani M, Almasi SZ, Amini M (2015). The product of triglycerides and glucose in comparison with fasting plasma glucose did not improve diabetes prediction. Acta Diabetol.

[55] Er LK, Wu S, Chou HH (2016). Triglyceride glucose-body mass index is a simple and clinically useful surrogate marker for insulin resistance in nondiabetic individuals. PLoS ONE.

[56] Zheng S, Shi S, Ren X (2016). Triglyceride glucose-waist circumference, a novel and effective predictor of diabetes in first-degree relatives of type 2 diabetes patients: cross-sectional and prospective cohort study. J Transl Med.

